# Applying the brakes to transcription: regulation of gene expression by RNA polymerase pausing

**DOI:** 10.1128/jb.00084-25

**Published:** 2025-06-06

**Authors:** Oshadhi T. Jayasinghe, Paul Babitzke

**Affiliations:** 1 Department of Biochemistry and Molecular Biology, Center for RNA Molecular Biology, Pennsylvania State University 219262, University Park, Pennsylvania, USA; Geisel School of Medicine at Dartmouth, Hanover, New Hampshire, USA

**Keywords:** transcription, RNA polymerase pausing, riboswitch, transcription attenuation, NusG, NusA, transcriptional regulation

## Abstract

Transcription by RNA polymerase is punctuated by transient pausing events. Pausing provides additional time for proper RNA folding and binding of regulatory factors to the paused transcription elongation complex or the nascent RNA. Depending on the organism and the genomic context, the general transcription elongation factors NusA and NusG stimulate or suppress pausing. Both *Escherichia coli* and *Bacillus subtilis* NusA stimulate pausing *in vitro*, while the genome-wide role of NusA on pausing has only been examined in *B. subtilis*. NusG-dependent pausing was identified throughout the *B. subtilis* genome, and in several instances, these pauses were shown to regulate the expression of the downstream gene(s). This pro-pausing activity was also observed for *Mycobacterium tuberculosis* NusG. In contrast, *E. coli* NusG functions as an anti-pausing factor by suppressing pausing throughout the genome. These differences in the function of NusG highlight the importance of studying fundamental processes in a variety of bacterial species. This review will highlight recent advances gained by the ability to identify pauses genome-wide that are either stimulated or suppressed by these two conserved transcription elongation factors.

## INTRODUCTION

**Fig 1:**
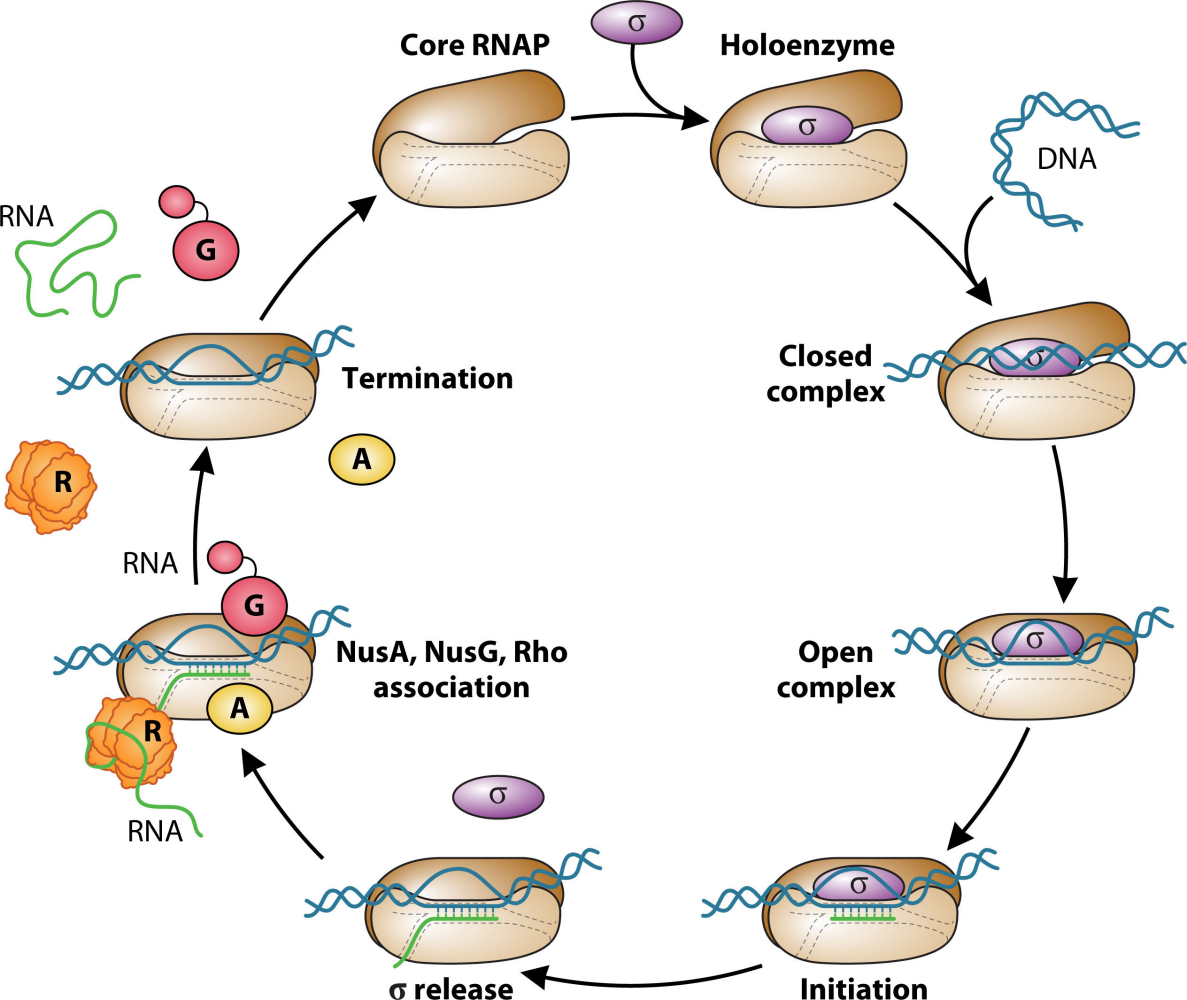
Model of the transcription cycle. The holoenzyme binds to the promoter and unwinds the DNA so that transcription can initiate. Once the RNA reaches about 15 nucleotides in length, the σ factor is released, which frees up the RNAP binding surfaces for NusA and NusG. Rho can also bind to RNAP so that the RNA enters the central hole of the Rho hexamer as it exits the RNAP exit channel.

Bacterial RNA polymerase (RNAP) is a multi-subunit enzyme that converts the stored information in DNA into RNA by transcription (1). As the first step of gene expression, transcription is carried out by RNAP acting in concert with numerous other auxiliary factors (2, 3). Bacteria can tune their transcriptional programs in response to changing environments through numerous mechanisms that regulate the activity of RNAP. In all organisms, transcription can be divided into three mechanistically and structurally distinct stages: initiation, elongation, and termination (Fig. 1). The regulation of different stages of the bacterial transcription cycle occurs through a medley of biochemical interactions between RNAP, DNA, nascent RNA, and trans-acting transcription factors (4–7). Promoter-specific initiation of transcription requires an additional subunit, σ, which binds the core RNAP to form the holoenzyme (8). Housekeeping σ factors (and alternative σ factors in specialized cases) guide RNAP through the essential steps of initiation: promoter recognition and opening and the synthesis of the first few nucleotides of the transcript (8, 9). Once the nascent RNA reaches a length of 13–15 nucleotides in which 9–10 nucleotides reside in an RNA-DNA hybrid with the template DNA strand, the transcription complex undergoes promoter clearance and transitions to the elongation phase with the concomitant release of the σ factor (7–9).

**Fig 2:**
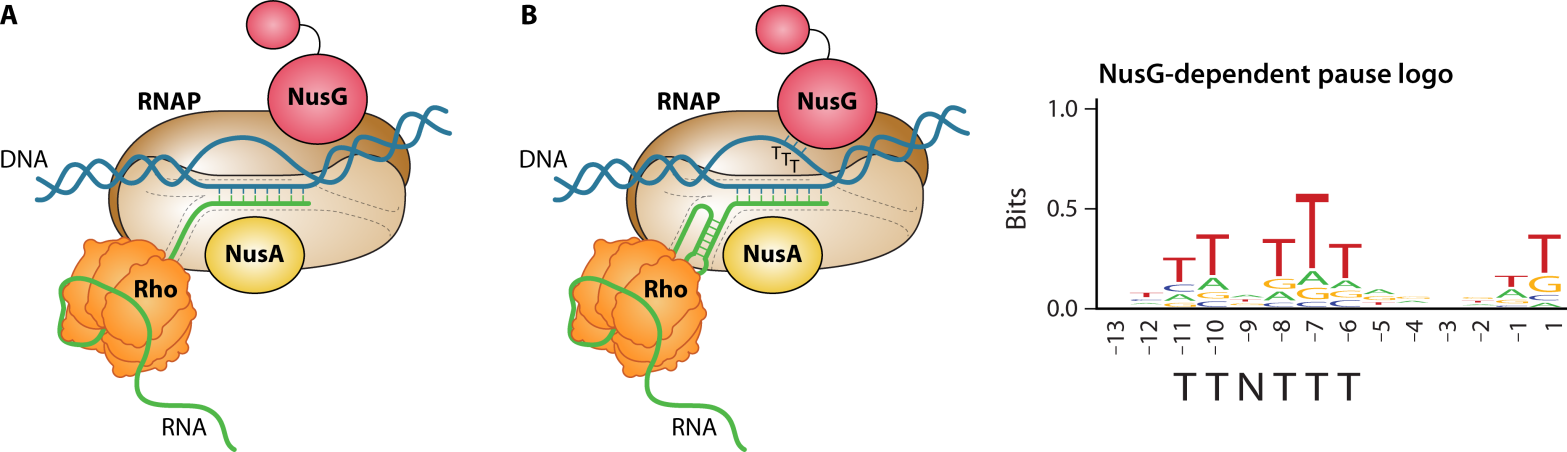
Models of transcription elongation complexes. (**A**) The transcription elongation complex is poised to recognize and respond to pause and termination signals. (**B**) NusG interacts with its TTNTTT pause motif in the non-template DNA strand. A pause hairpin forms in the RNA exit channel with the assistance of NusA, leading to a stabilized paused EC. NusA also assists with the formation of intrinsic terminator hairpins in the RNA exit channel. The TTNTTT sequence logo of NusG-dependent pause sites is shown with −1 representing the 3′ end of paused RNA.

The transcription elongation complex (TEC) is highly stable and capable of uninterrupted synthesis of RNA chains thousands of nucleotides long with high processivity by incorporating about 50–100 nucleotides per second (10, 11). Several transcription factors bind directly to the TEC (12). The cross talk between elongation factors, RNAP, and other components of the TEC modulates the transcription activities of the TEC by recognizing and responding to a variety of regulatory signals (12). NusA and NusG are two transcription factors that bind to RNAP during elongation (12) (Fig. 2). By doing so, these two factors regulate the processivity of RNA synthesis negatively or positively, depending on the organism and the genomic context (12–19). For example, in *Escherichia coli*, NusG increases transcription processivity by ensuring that the clamp of RNAP remains closed around the DNA binding channel and stabilizing the upstream fork junction where the upstream double-stranded DNA and the transcription bubble meet, thus ensuring that RNAP remains in an RNA synthesis competent state (15). The anti-pausing activity of *E. coli* NusG suppresses thousands of pauses throughout the genome (17, 19). In contrast, NusG is a pause-promoting factor in *Bacillus subtilis* (16, 18). NusA works similarly in *E. coli* and *B. subtilis*. When bound to RNAP, NusA facilitates the formation of RNA hairpins in the RNA exit channel of RNAP, thereby affecting pausing and intrinsic termination (13, 14, 20).

Transcription elongation is terminated when RNAP encounters a termination signal, resulting in the release of the nascent transcript through its RNA exit channel (21). Transcription termination typically occurs via two common mechanisms in bacteria. One mechanism is intrinsic termination, where formation of a characteristic terminator hairpin in the nascent RNA promotes transcript release (21, 22). Intrinsic terminators consist of a GC-rich hairpin that forms within the RNA exit channel of RNAP, which is followed by a U-rich tract (23, 24). This hairpin induces the melting of the U:A-rich RNA:DNA hybrid that is accommodated within the main channel of RNAP, leading to transcript release and dissociation of TEC components from one another (23–25). The second termination mechanism, known as Rho-dependent termination, requires the activity of Rho, which is a homohexameric ATPase with RNA translocase and RNA:DNA helicase activities (26–28). Rho-dependent termination occurs when Rho contacts a specific RNA element known as the Rho utilization (*rut*) site and a paused RNAP (29–31). Upon binding to a *rut* site, Rho induces an allosteric structural transition in RNAP such that it enters a catalytically inactive state and dislodges the RNA from the active site (32, 33). Although intrinsic termination suggests the lack of factor involvement and was in fact called Rho-independent, recent work in *B. subtilis* has shown that NusA, NusG, and Rho have prominent roles in regulating intrinsic termination (34–36).

Through a genome-wide mapping strategy to identify authentic 3′ ends called Term-seq, ~1,600 intrinsic termination events were identified *in vivo* in *B. subtilis* (34–36). In conjunction with an engineered NusA depletion strain, the genome-wide effect of NusA on termination was determined (34). NusA promotes hairpin formation and regulates proper termination, especially at suboptimal terminators with weak non-canonical hairpins and/or interrupted U-tracts (34). Hence, depletion of NusA caused global misregulation of gene expression via readthrough of suboptimal terminators (34). The role of NusG in intrinsic termination was also determined, which involves its ability to stimulate RNAP pausing at the point of termination (35, 36). The role of Rho in intrinsic termination occurs via a passive mechanism in which Rho prevents the formation of antiterminator-like RNA structures that could otherwise compete with the terminator hairpin downstream of coding sequences (36, 37).

## TRANSCRIPTIONAL PAUSING BY RNAP

Upon the development of techniques that could visualize specific nascent RNA products, it was evident that RNA synthesis by RNAP was discontinuous (38, 39). Early observations via gel electrophoresis of the accumulation and disappearance of intermediary RNA transcripts of discrete lengths during *in vitro* transcription indicated that RNAP transiently paused at specific sites during transcription (38, 40). The advances afforded by the development of native elongating transcription sequencing (NET-seq) demonstrated that transcription is occasionally punctuated by elemental and regulated pausing events upon encountering specific signals (41). Pause signals within the nascent RNA and/or DNA cause reversible isomerization of RNAP into a state that inhibits elongation (41–43). In this elemental pause state, RNAP transiently rearranges into a catalytically inactive conformation that is unable to load NTP substrate (43, 44). Entry into elemental pause states is a fundamental property of RNAP (44, 45).

Several factors contribute to RNAP pausing during transcription (41, 46–49). A fundamental feature of pausing is the rearrangement of the TEC into a paused EC (PEC) due to the kinetic competition between the paused state and a TEC that bypasses a pause site (10). The conformational fluctuations of RNAP during transcription help explain the PEC. During elongation, the TEC remains dynamic, thereby maintaining the ability to fluctuate among multiple conformations. Conformational changes in different domains of RNAP are needed for its transcriptional activity (50–52). Transcript extension is achieved by the addition of one nucleotide at a time through the nucleotide addition cycle (NAC). The sequence of steps of the NAC is repeated with each NTP addition. This cycle includes translocation of the 3′ end of the RNA from the pre- to the post-translocation register, which vacates the binding site for the incoming NTP, aligning the next template DNA base for base pairing, NTP binding with Mg^2+^ at the active site, folding of the trigger loop (TL) into trigger helices (TH), and phosphodiester bond formation (53, 54). Subsequent unfolding of the TH, release of pyrophosphate, and forward translocation prepare the active site for the next NAC (53, 54). Movements of the domains, loops, and modules of RNAP accompany each step of the NAC, which are critical for the continuation of transcript elongation (54). At pause sites, the NAC is interrupted at one or more steps via effects of RNA and DNA sequences on RNAP conformation. Other factors that contribute to transcriptional pausing do so by facilitating and stabilizing off-pathway states (55).

Transcriptional pauses are thought to begin with the isomerization of the EC into a transient, catalytically inactive elemental paused state (44). Elemental pausing occurs every 100–200 nucleotides on average, which involves a limited *in vivo* consensus sequence (43, 44). The multipartite elemental pause signal comprises sequences at the upstream fork (G_-10_, relative to the RNA 3′ end at position −1), RNA-DNA hybrid, downstream fork (Y_-1_ G_+1_), and downstream duplex DNA, and together these elements affect pause duration (43, 45, 56, 57). The majority of elemental pauses are short-lived and, therefore, are not considered of regulatory significance (43). However, elemental pauses can serve as precursors for stabilized pauses. An elemental PEC (ePEC) can further rearrange into long-lived pause states due to other processes that contribute to pause stabilization. One such process is the backtracking of RNAP. Backtracking occurs via backward movement of RNAP, which causes the 3′ end of the nascent RNA to move from the active site into the secondary channel (48, 58). Backtracked complexes can be rescued by the transcript cleavage factors GreA/GreB (59). These factors stimulate the intrinsic hydrolyzing activity of RNAP, which removes the 3′ extruded portion of the transcript to generate a new RNA 3′ end in the catalytic site, thereby reactivating the EC (59). Pauses can also be stabilized by the formation of RNA secondary structures, such as hairpins in the RNA exit channel, that interact with and modify the ePEC conformation (46), by the interaction of transcription regulatory factors (14, 16, 18, 60), or a combination of these mechanisms (14, 16, 41, 49, 60–62). Stabilized pauses at regulatory sites allow synchronization of the position of RNAP on the DNA template with co-transcriptional RNA folding and/or regulatory factor binding, orchestrating regulatory events that modulate gene expression (14, 16, 18, 60–62).

Formation of an RNA duplex 11–12 nt upstream from a paused RNA 3′ end of an ePEC can increase the longevity of the pause by ~10-fold (63). Cryo-EM structures of *E. coli* PECs indicate that the pause hairpin is in the RNA exit channel (20, 64). The inner wall of the RNA exit channel is lined with positively charged amino acid residues, presumably facilitating the formation of an RNA hairpin within the channel (20, 64). The spacing between the 3′ end of the paused transcript and the RNA hairpin in the RNA exit channel determines the interactions of the hairpin with the flap domain of RNAP (64, 65). Hairpin-stabilized pause signals may include specific RNA/DNA sequences and interactions with transcription elongation factors (14, 16, 60–62).

Transcriptional pauses stabilized by transcription factors have recently become a compelling topic of interest due to the regulatory intricacy that they add to the fundamental process of pausing. For example, a transcription factor that stabilizes a specific pause may take part in other molecular events such as recruiting a second regulator to the TEC or RNA, inhibiting the activity or binding of a second regulator to the TEC or RNA, and rearrangement of RNA or RNAP. By doing so, pausing can be synchronized with a cascade of events that are leveraged in co-transcriptional regulatory mechanisms of gene expression (14, 16, 18, 61, 62, 66). In this review, we are highlighting the role of general transcription elongation factors NusA and NusG in pausing and the regulatory consequences of factor-dependent pausing.

## TRANSCRIPTION ELONGATION FACTOR NusA

NusA is a highly conserved, multi-functional transcription elongation factor that is essential for the viability of both gram-negative *E. coli* and gram-positive *B. subtilis* (34, 67). NusA was first discovered as a factor required for protein N-mediated antitermination of bacteriophage λ, along with other N-utilizing substance (Nus) factors, including NusG, NusB, and NusE (also known as ribosomal protein S10) (68). NusA was also shown to be necessary for antitermination of rRNA synthesis (69) and the efficient expression of other genes such as *lacZ* (70). This transcription factor is a multi-domain protein with an N-terminal domain (NTD), RNA binding domains S1, KH1 and KH2, and a C-terminal domain (CTD) that varies between *E. coli* and *B. subtilis* (71). In *E. coli*, NusA contains a large CTD with two acidic repeat domains (AR1 and AR2) that interact with other factors. This large CTD is missing in *B. subtilis* NusA (71, 72). Following the release of the σ factor, the NTD of NusA binds to the TEC via interactions with the flap-tip helix of the β subunit flap domain of RNAP (20). Once bound, it can directly interact with RNA using its S1, KH1, and KH2 domains (20). By binding adjacent to the RNA exit channel of RNAP, the NusA NTD provides an additional set of positively charged residues, extending the cavity, which facilitates nucleation and stabilization of RNA hairpins in the RNA exit channel (20). Upon interaction with RNAP, a structural rearrangement of NusA leads to an allosteric repositioning of conserved basic residues that could enable their interaction with an RNA pause hairpin, thereby stabilizing the paused complex (73). Moreover, binding of NusA results in an allosteric widening of the RNA exit channel, which acquires the ability to readily accommodate an RNA hairpin duplex and a single-stranded RNA segment (20). Structural and biochemical studies of *E. coli* PECs revealed that an RNA hairpin-stabilized pause can be further prolonged 10-fold by NusA (20, 63). Thus, when bound to RNAP, NusA can facilitate the formation of an RNA hairpin in the RNA exit channel (Fig. 2B), a property that allows this factor to serve as both a pausing and a termination factor (14, 34, 46).

NusA has been known to stimulate pausing of *B. subtilis* and *E. coli* RNAP *in vitro* for many years (14, 61, 74–77). NusA-stimulated pausing was first discovered during studies of the *E. coli trp* operon attenuation mechanism (75–77). In *B. subtilis*, NusA was initially shown to stimulate pausing at two positions in the *trp* operon leader region (14, 61, 74, 78). The first pause provides additional time for the binding of tryptophan-activated TRAP protein, thereby promoting termination through a transcription attenuation mechanism *in vitro* (14, 74). The second pause participates in a *trpE* translation repression mechanism. In this case, pausing provides time for binding of tryptophan-activated TRAP such that bound TRAP promotes the formation of a secondary structure in the *trpE* leader region that sequesters the Shine-Dalgarno sequence, thereby blocking ribosome binding (74, 78). Single-molecule experiments with *E. coli* RNAP provided additional insight into NusA-stimulated pausing (79). In this study, NusA was shown to increase the probability of entering into short- and long-lifetime pauses, while decreasing the overall elongation rate of RNAP. Of particular interest, NusA from *B. subtilis* and *E. coli* can substitute for one another in stimulating pausing *in vitro* (61), suggesting that they both act through similar mechanisms.

## TRANSCRIPTION ELONGATION FACTOR NusG

NusG is another transcription elongation factor that binds to the TEC after dissociation of the σ factor (12). NusG is the only universally conserved transcription factor, with its archaeal and eukaryotic homolog known as Spt5 (80). Bacterial NusG is composed of an N-terminal (NGN) domain and a C-terminal KOW domain, which are connected by a flexible linker (15, 81). Regions of RNAP that interact with NusG overlap with the σ factor binding region, suggesting that NusG binds directly to RNAP after σ release (82). During transcription elongation, the NGN domain binds to the clamp helices of the β′ subunit of RNAP, while the KOW domain is free to interact with various regulatory partners (15). For example, in *E. coli,* the KOW domain interacts with Rho termination factor or ribosomal protein S10, enabling NusG to act as a regulator of transcription polarity and coupling of transcription and translation, respectively (83, 84). Moreover, *E. coli* NusG interacts with the S1 domain of NusA when coordinated by NusE and the λ N protein in N-mediated antitermination (80). *E. coli* NusG functions as a transcription processivity factor via its ability to ensure that RNAP remains in a nucleotide incorporation-competent state (85). In addition, *E. coli* NusG suppresses pausing by stimulating forward translocation and preventing backtracking of RNAP (86). However, novel functions were revealed for *B. subtilis* NusG through genome-wide investigation of transcriptional pausing and termination (16, 18, 35, 61, 62, 74).

## RNET-SEQ AND IDENTIFICATION OF GENOME-WIDE RNAP PAUSING *IN VIVO*

Several lines of evidence had pointed toward roles for NusA and NusG in transcriptional pausing. Both NusA and NusG were known to dramatically affect RNAP pausing *in vitro* (14, 18, 61, 62, 74, 87). In addition, structural analysis of an *E. coli* PEC with NusA revealed that NusA could serve as a pausing factor (13, 20, 73). However, the genome-wide pausing behavior of RNAP in a NusA- and/or NusG-dependent manner had not been identified due to technical challenges. A crucial *in vivo* RNA sequencing method that can capture the nascent RNA 3′ ends that are protected in the active site of RNAP with high resolution has not been developed. In addition, NusG is essential in *E. coli,* while NusA is essential in both *E. coli* and *B. subtilis.* The essential nature of these proteins prevented the generation of loss-of-function mutants to determine their roles in pausing and gene regulation.

The first step in overcoming these barriers was the construction of a viable *B. subtilis* NusA depletion strain (34). In this strain, NusA was solely produced exogenously via an IPTG-inducible promoter (34). Growth of this strain in the presence of IPTG resulted in near wild-type (WT) levels of NusA, while withholding IPTG from the growth media led to depletion of NusA to about 2% of WT levels (34).

**Fig 3:**
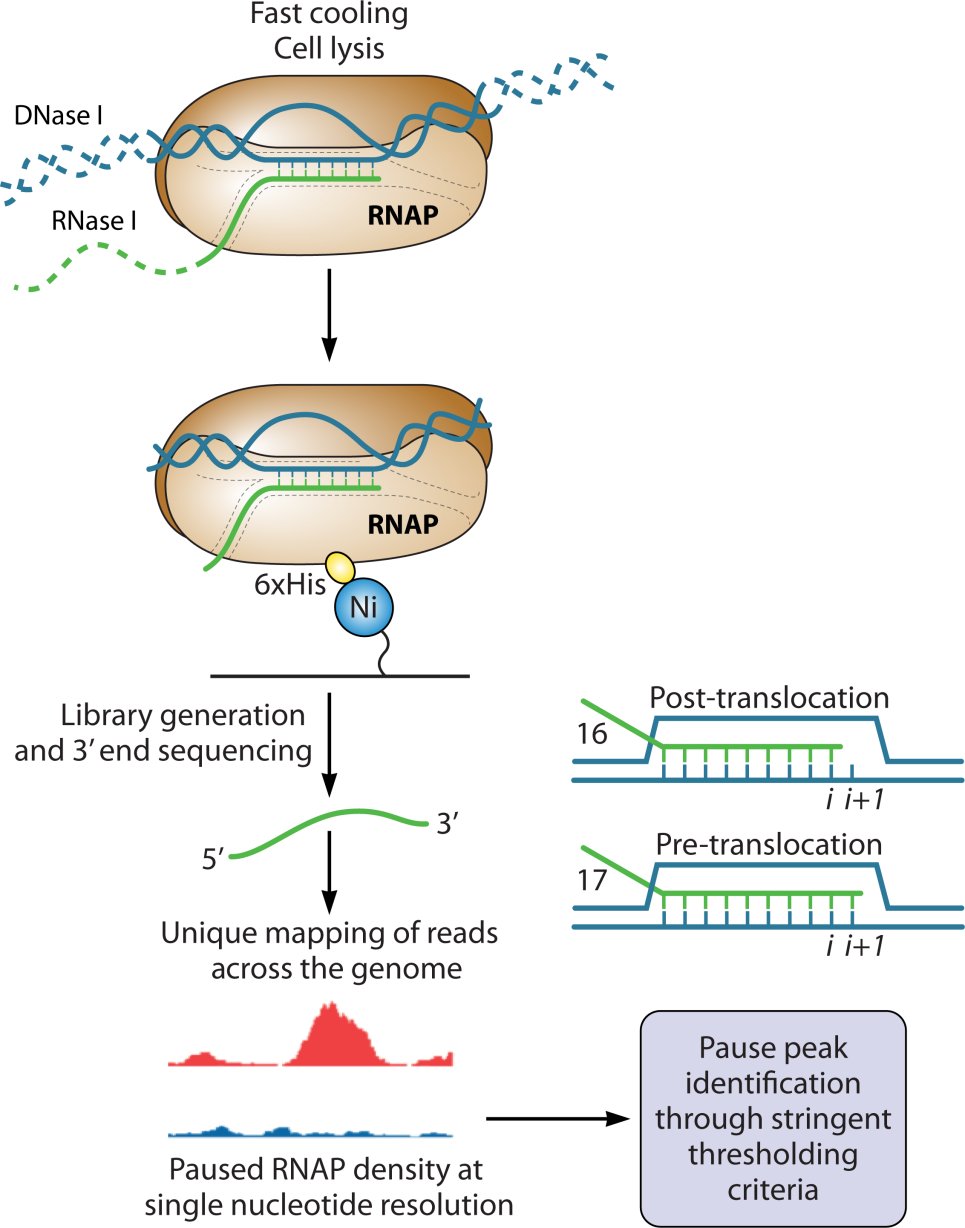
Schematic diagram of RNET-seq. Cell culture samples are collected at an appropriate time and flash frozen. Following cell lysis, the samples are treated with RNase I and DNase I. RNAP is then purified by a His tag along with the RNA and DNA that is protected by RNAP. The short RNAs are sequenced following library preparation. Pause peaks are identified computationally. The translocation states identified by RNET-seq are also shown. Following nucleotide addition, the RNA length is 17 nucleotides (pre-translocation register). In the post-translocation register, the RNA length is 16 nucleotides, and the i + 1 site is available for NTP binding.

The next pivotal stride was the development of RNET-seq, which is a robust method to identify paused RNAP throughout the genome (Fig. 3) (16, 88). RNET-seq combines NET-seq with RNase I digestion to degrade the exposed 5′ end of nascent RNA and any contaminating released RNA. This method allows mapping of authentic nascent RNA 3′ ends at single-nucleotide resolution. Moreover, this method enables probing of the translocation state of RNAP at pause sites by determining the length of the RNAP footprint on nascent RNA (Fig. 3). Precise computational identification of pause peaks in RNET-seq data encompasses stringent selectivity criteria of parameters in mapped data (16, 60). By obtaining a comprehensive set of transcriptome-wide pauses and comparing such data sets across different strains/biological conditions, one can identify the transcriptional pause positions that are affected by factors or conditions *in vivo.* The work described in the subsequent sections originated from a wealth of data generated by RNET-seq in WT, *nusA* depletion (*nusA*_dep_), *nusG* deletion (Δ*nusG*), and *nusA*_dep_ Δ*nusG* strains to examine the NusA- and NusG-dependent RNAP pausing behavior in *B. subtilis.*

## TRANSCRIPTOME-WIDE EFFECTS OF NusA ON RNAP PAUSING

Genome-wide mapping of nascent RNA 3′ ends using RNET-seq identified the effects of NusA on transcriptional pausing in *B. subtilis* (60). NusA exhibited modest effects on pausing, with pause-stimulating and pause-suppressing effects genome-wide. Based on the pause identification thresholding criteria used in the RNET-seq study, NusA stimulated pausing at 129 positions in 93 different genes. By comparison, 83 NusA-suppressed pause peaks were identified in 57 genes. *In silico* RNA folding analysis of the pause regions identified putative pause-stabilizing hairpins for 67% of the NusA-stimulated pauses, suggesting that RNA hairpins are a common component of the NusA-stimulated pause signals. These hairpins were positioned between 10 and 13 nucleotides upstream from the pause position, and this hairpin-to-3′ end distance is similar to what was observed for known hairpin-stabilized pauses (62, 87, 89, 90). However, a consensus NusA-stimulated pause motif was not identified in the DNA or RNA sequences surrounding the pause sites. *In vitro* pausing assays with purified recombinant NusA showed that NusA stabilizes pausing *in vitro* at several pause sites identified *in vivo*. The relative density of NusA-stimulated pause peaks in 5′ leaders is far higher than in open reading frames (ORFs), suggesting that NusA could be an important participant in transcription attenuation or translational control mechanisms. In fact, of the NusA-stimulated pauses within 5′ leaders, two-thirds were located upstream of known transcription attenuators.

## EFFECTS OF NusA-STIMULATED PAUSING IN MODULATING TRANSCRIPTION

A few studies on how NusA-stimulated pausing affects riboswitch-based transcription regulation demonstrated that the time window for ligand sensing is widened in the presence of NusA, thereby allowing a more efficient gene regulatory response (91–93). In the case of the *B. subtilis ribD* riboswitch, the addition of NusA increased transcription termination while decreasing the *T*_50_ of FMN (i.e., the half-effective FMN concentration for increased termination) (92). Kinetic characterization of the *B. subtilis* adenine-responsive riboswitch controlling *pbuE* transcription showed that NusA improved riboswitch function (93). In *pbuE*, NusA decreased the overall transcription rate and decreased the *T*_50_ (93). *In vitro* transcription experiments of the *E. coli thiC* riboswitch showed that three major pauses in the riboswitch are affected by NusA, with a hairpin stabilization at the most upstream pause (91). Destabilization of the pause hairpin resulted in a weaker pause, a decreased effect of NusA on expression, as well as decreased TPP binding affinity to the full-length riboswitch (91).

Proper co-transcriptional folding of RNA into biologically relevant structures must avoid the formation of undesirable structures, and specific pausing can alter and guide RNA folding (94, 95). *In vitro* transcription experiments using *E. coli* RNAP and NusA showed that NusA accelerated the folding of the ribozyme of *B. subtilis* RNase P (94). The effect of NusA was attributed to the enhancement of pausing because NusA did not accelerate folding using a mutant RNAP that failed to pause or respond to NusA (94). Single-molecule studies using a reconstituted TEC revealed an interactive mechanism where a dynamic competition between NusA and a ligand modulated a riboswitch. In this F^−^-responsive riboswitch from *Bacillus cereus,* binding of F^−^ stabilized an RNA pseudoknot, thereby preventing the formation of an intrinsic terminator hairpin, leading to transcription of the downstream gene. NusA decreased the overall rate of transcription, which promoted the formation of the terminator hairpin that is antagonized by F^−^ binding. The cotranscriptional formation of the pseudoknot accelerated the release of NusA from the TEC and thereby modulated the transcription rate (96).

## TRANSCRIPTOME-WIDE EFFECTS OF NusG ON RNAP PAUSING

NusG-dependent RNAP pausing was first identified *in vitro* at two positions in the *B. subtilis trpEDCFBA* operon 5′ leader region; pausing at both positions is also stimulated by NusA (61, 74, 78, 87). These pauses were found to participate in transcription attenuation and translation repression mechanisms by providing additional time for binding of tryptophan-activated TRAP protein to the nascent transcript (61, 74, 78, 87). The mechanism of NusG-dependent pausing was found to involve interaction of NusG with a T-rich sequence motif in the non-template DNA (ntDNA) strand of the paused transcription bubble, as well as an RNA pause hairpin 11–12 nt upstream of the RNA 3′ end (18, 74).

RNET-seq studies in WT and Δ*nusG* strains identified 1,600 strong NusG-dependent pause sites throughout the *B. subtilis* genome. Bioinformatic analysis of the NusG-dependent pause site sequences revealed a conserved consensus T-rich pause motif that is necessary, but not sufficient, for NusG-dependent pausing (16). The consensus NusG-dependent pause motif is 5′_-11_TTNTTT_-6_ 3′ in the ntDNA strand relative to the 3′ end of the paused RNA at position −1 (Fig. 2B) (16, 18). Biochemical studies revealed that the NGN domain of NusG makes sequence-specific contacts with this motif (16). The NusG-dependent pauses identified by RNET-seq included the pause site in the *trpEDCFBA* operon leader involved in the repression of *trpE* translation but not the site associated with attenuation (16). About 20% of the 1,600 pause sites are located within 5′ leader regions, with the remaining 80% in ORFs. Several of the pauses in 5′ leader regions were subsequently shown to participate in transcription attenuation and/or translation attenuation mechanisms (see below). However, the roles of pausing in ORFs are not known.

NusG binds to TECs with relatively low affinity such that RNAP-bound and free NusG can readily exchange (18). Thus, it was proposed that the sequence-specific interaction with the pause motif increases the affinity of NusG specifically for a TEC containing a TTNTTT motif in the transcription bubble (18). A cluster of surface-exposed amino acid residues of NusG was identified that includes the critical N81 and T82 residues that recognize the T-rich motif (18). *E. coli* NusG has S85 and V86 at these positions and does not stimulate pausing at the T-rich motif, further confirming the requirement of specific interactions between NusG residues and the pause motif (18). However, only a small fraction of TTNTTT sequences present in the *B. subtilis* genome stimulate strong RNAP pausing *in vivo* (16). Therefore, this motif is not sufficient for NusG-dependent pausing in *B. subtilis.* Moreover, binding of *B. subtilis* NusG to *E. coli* RNAP did not stimulate pausing in the *trp* leader (61), suggesting that additional elements are required for the NusG-dependent pausing mechanism.

## STRUCTURAL ANALYSIS OF THE NusG-DEPENDENT PAUSING MECHANISM

Cryo-EM structural studies of the NusG-dependent *M. tuberculosis* PEC revealed that the interaction of NusG with the ntDNA strand rearranges the transcription bubble such that three consecutive T residues of the pause motif are extruded and sandwiched in a cleft between NusG and the β lobe of RNAP (66, 97). Structures from one of these studies revealed a detailed allosteric mechanism of transcription inhibition (66). A key conformational change in all cellular RNAPs needed for RNA synthesis is TL folding (53). This conformational change is directly linked to an intrinsic motion of RNAP known as the swivel module rotation, where the cleft between NusG and the β lobe is widened (swiveled) and narrowed (non-swiveled) (66). Interestingly, the NusG-ntDNA interaction at a pause site traps RNAP in the swiveled conformation, thereby allosterically preventing TL folding and inhibiting NTP incorporation (66).

## NusG-DEPENDENT PAUSING IN POST-TRANSCRIPTION INITIATION REGULATORY MECHANISMS

NusG promotes pausing about once every 3 kb in the *B. subtilis* genome (16). Initially, four NusG-dependent pause sites in 5′ leaders were characterized and shown to be involved in regulating the expression of the downstream gene. As described above, a NusG-dependent pause participates in a translation repression mechanism where it provides time for tryptophan-activated TRAP to bind to the nascent *trp* transcript, promoting the formation of an RNA hairpin that sequesters the *trpE* Shine–Dalgarno sequence (61, 74, 78, 87). NusG-dependent pausing in the *tlrB* leader provides time for translation of a short leader peptide, which is vital for tylosin-dependent *tlrB* expression through an intricate mechanism involving transcription attenuation and translation attenuation (62). In addition, NusG-dependent pauses identified through RNET-seq in the *ribDEAHT* and *vmlR* leaders regulate gene expression. Pausing in the *ribDEAHT* leader occurs within the *ribD* riboswitch that binds the flavin nucleotides FMN and FAD (16). Binding of FMN to the nascent RNA results in termination (attenuation) of transcription upstream of the start codon of *ribD*. NusG-dependent pausing at this site provides additional time for co-transcriptional binding of FMN to the nascent RNA, resulting in increased termination in the *ribD* leader region (16). The NusG-dependent pause in the *vmlR* leader plays a critical role in regulating the expression of the ABCF antibiotic resistance factor VmlR in an antibiotic-dependent manner. As for *tlrB*, this complex regulatory mechanism includes transcription attenuation and translation attenuation mechanisms (98).

Out of the 1,600 NusG-dependent pause sites identified *in vivo* through RNET-seq, about 20% are located in 5′ leader regions, while the remaining pause sites are mapped to ORFs (16). Considering that 5′ leaders comprise less than one-tenth of the genome of this organism, this distribution indicates an enrichment of pauses in 5′ leaders, pointing toward the possibility for more regulatory roles for NusG. Driven by this observation, a recent investigation identified a regulatory role of several more NusG-dependent pauses. The precise identification of the *in vivo* NusG-dependent pause sites, the pause signals, and elements of the pausing mechanism (16, 66) laid the groundwork for further investigation into the regulatory consequences of pauses stabilized by NusG.

## NusG-DEPENDENT PAUSING IS A COMMON REGULATORY FEATURE IN RIBOSWITCH MECHANISMS

In bacteria, the expression of certain genes is controlled by riboswitches, which are structured regulatory domains embedded within the 5′ leader region of mRNAs that directly sense specific intracellular metabolites (99–104). The aptamer domain of a riboswitch serves as a binding pocket for a specific ligand, whereas the expression platform undergoes structural transitions in response to ligand binding, modulating the expression of the downstream gene (99–101, 104–107). This ligand-induced interconversion of structural states can regulate gene expression via one of several distinct mechanisms, including transcription attenuation, antitermination of transcription, and repression or activation of translation initiation (104, 108–113). In addition to the *ribD* riboswitch described above, several NusG-dependent pauses associated with known and predicted riboswitches were identified (16, 114). The pauses in the *fmnP*, *tenA*, *mgtE*, *lysP,* and *mtnK* riboswitches are located in positions preceding the critical decision between the formation of alternative antiterminator or terminator structures, which is a critical feature of transcription attenuation mechanisms (114). A recent investigation of the regulatory effects of these pauses in the riboswitch mechanisms revealed important functions for NusG in ligand-dependent transcription attenuation (114). *In vitro* transcription assays confirmed the presence of the NusG-dependent pause sites that were identified by RNET-seq (16, 114).

In the FMN-sensing *fmnP* riboswitch, NusG-dependent pausing participates in a previously unrecognized transcription attenuation mechanism to control the expression of the downstream riboflavin transporter (114). Pausing increased the termination efficiency in response to high FMN concentrations *in vitro.* Expression of chromosomally integrated WT and pause-defective transcriptional fusions showed that the pause reduced the expression of *fmnP* in response to FMN *in vivo*. Elimination of the pause resulted in weak attenuation in response to FMN, demonstrating the critical regulatory role of the pause in this system. Pausing in this riboswitch did not affect the previously reported translational control mechanism that also controls *fmnP* expression. This differential response to pausing is due to the mechanistic differences between transcriptional and translational riboswitches. Transcriptional riboswitches rely on a restricted time window for ligand association before completion of the terminator, whereas translational riboswitches can associate with the ligand multiple times within its lifetime co- or post-transcriptionally (92, 93, 115). Thus, pausing fulfills a crucial role for efficient attenuation by modulating transcriptional kinetics to facilitate ligand association in this riboswitch.

NusG-dependent pausing in the TPP-binding *tenA* riboswitch participates in modulating the expression of thiaminase II, an enzyme involved in the thiamine salvage pathway (114). Pausing in this riboswitch increased termination at the attenuator *in vitro* and influenced the TPP-mediated transcription attenuation *in vivo* (114). Compared to the other riboswitches that were investigated, pausing played a more limited role in the modulation of the *tenA* riboswitch. TPP binds to the *tenA* aptamer with exceptionally high affinity (101, 102, 116). Thus, it was proposed that the high affinity between TPP and the aptamer may be responsible for the more limited role that pausing has on regulating the expression of this riboswitch (114).

NusG-dependent pausing in the *mgtE* riboswitch helps to maintain Mg^2+^ homeostasis by regulating the expression of a Mg^2+^ transporter (114). In this system, pausing increased termination in response to Mg^2+^ *in vitro*, and the elimination of pausing resulted in much weaker Mg^2+^-dependent attenuation *in vivo*. Together, these results established that NusG-dependent pausing allows fine-tuning of Mg^2+^ transport. A predicted lysine-responsive riboswitch was identified in the *lysP* leader region. NusG-dependent pausing participates in a lysine-dependent transcription attenuation mechanism that controls the expression of LysP, a lysine transporter (114). In the SAM-binding S-box riboswitch of *mtnK*, NusG-dependent pausing participates in a SAM-dependent transcription attenuation mechanism to regulate the methionine salvage pathway (114). In this riboswitch, pausing occurs prior to completion of the entire SAM aptamer. RNA structure folding predictions of the *mtnK* leader suggested that pausing would facilitate co-transcriptional folding of the RNA to form the SAM-binding competent aptamer (114).

Based on the reported examples, NusG-dependent pausing allows RNAP to respond to a variety of intracellular signals, resulting in proper transcriptional readout for changing environmental and/or growth conditions. The function of transcriptional riboswitches is kinetically controlled by the rate of transcription, co-transcriptional folding of the RNA, and the affinity of ligand binding (92, 117–119). Isolated aptamers have shown nanomolar *K*_*D*_ values for their ligand binding, although they require a much higher ligand concentration to effectively control gene expression (92, 120). Transcription occurs at about 50–100 nucleotides per second, and to surpass the kinetics of transcription, the cellular concentrations of ligands must be much greater than their *K*_*D*_ value to ensure sufficiently rapid binding (92, 117, 118). If the aptamer lacks enough time to equilibrate with the environment, the RNA could commit to an alternative folding pathway that is irreversible (121, 122). Based on the measured association kinetics of ligand binding in riboswitches, continued transcription at a normal rate would reduce the time window for ligand association (91, 123, 124). A programmed pause at these sites can provide additional time for ligand binding and structural rearrangement of the RNA (91, 92, 114, 125–131).

## PERSPECTIVES

NusA and NusG are conserved transcription elongation factors. Even though their role in transcriptional pausing was inferred based on their observed *in vitro* functions, experimental challenges restricted our understanding of how these factors affected the intracellular genome-wide pausing behavior of RNAP. Thus, the regulatory significance of these factors in modulating gene expression via transcriptional pausing throughout the genome was unexplored. Recent technical advances helped overcome these barriers and laid the groundwork for the work identifying genome-wide effects of these factors on pausing and their regulatory significance.

NusA is universally conserved among bacteria and plays diverse roles in a context-dependent manner. In *E. coli*, NusA enhances intrinsic termination (132), facilitates co-transcriptional RNA folding into biologically relevant structures (94), participates in transcription-translation coupling (133–135), participates in transcription-coupled DNA repair (136), and extends the lifetime of hairpin-dependent pauses *in vitro* (49). NusA is an essential factor in both *E. coli* and *B. subtilis.* Harnessing *B. subtilis* as an alternative model organism to examine transcription and the successful construction of the NusA depletion strain made it possible to elucidate novel intracellular functions for this factor *in vivo* for the first time. In *B. subtilis*, expression of NusA is autoregulated by a transcription attenuation mechanism where NusA-stimulated intrinsic termination in its 5′ leader controls the extent of transcription into the gene (34). Thus, cellular NusA levels are tightly controlled, consistent with its role in critical cellular functions. NusA stimulates termination at about 75% of the intrinsic terminators in *B. subtilis* and prevents misregulation of global gene expression (34). High-resolution genome-wide mapping of RNAP, combined with the ability to investigate the effects of NusA depletion, opened the door to investigating the genome-wide role of this factor in transcriptional pausing (60).

NusG is the only transcription factor conserved across all three domains of life. NusG functions as a genome-wide pausing and intrinsic termination factor in *B. subtilis* (16, 35, 36). The density of NusG-dependent pause sites is threefold higher in untranslated regions (16), suggesting that pausing could regulate the expression of hundreds of genes in *B. subtilis* after transcription initiates. Pausing also plays an important role in NusG-stimulated intrinsic termination; deletion of NusG results in the misregulation of global gene expression and altered cellular physiology (35). At NusG-dependent pause sites, a high fraction of RNAP is in the post-translocation register in which RNAP has translocated forward by one base pair after nucleotide incorporation, resulting in a vacant active site for the incoming NTP (16). When not promoting pausing in a sequence-specific manner, *B. subtilis* NusG facilitates RNAP processivity by serving as a positive translocation factor, likely via a similar mechanism as *E. coli* NusG (16).

A phylogenetic analysis of bacterial NusG homologs was performed based on the presence of conserved amino acid residues that interact with the TTNTTT pause motif (16, 66). This analysis indicated that the *B. subtilis* type of pro-pausing NusG is widely conserved in bacteria, whereas the *E. coli* type of anti-pausing NusG is restricted primarily to γ-proteobacteria (16, 66). Thus, phylogenetic analysis suggests that NusG-dependent pausing is a widespread mechanism in bacteria. This pause-promoting function of NusG is consistent with the well-established role of its eukaryotic homolog Spt5 in promoter-proximal pausing. Eukaryotic Spt5 protein stimulates promoter-proximal pausing of RNAP II by interaction with negative elongation factor NELF (137). In addition, yeast Spt5 interacts with the ntDNA strand within the transcription bubble (138). Moreover, phylogenetic studies also revealed occasional horizontal transfer of *nusG* genes between bacterial and archaeal species (16). Therefore, it is likely that NusG/Spt5-dependent transcriptional pausing is a widely conserved mechanism. Although studies with *E. coli* have provided a wealth of information on the mechanism of transcription, the different activities of NusG/Spt5 that have been discovered in other organisms highlight the importance of studying cellular processes in a variety of species.

Based on the characterized examples, pausing in 5′ leaders is important for sensing intracellular signals to modulate transcription and/or translation. Transcriptional pauses provide additional time for these regulatory decisions. Thus, the correct position and the time provided by pausing are critical for synchronizing regulatory signals with transcriptional or translational readout (14, 16, 18, 61, 62, 74, 78, 87, 98, 114, 125, 139). Stabilized pausing may aid the timely recruitment of other regulators, guide nascent RNA folding, oppose or permit ribosome recruitment, and permit termination.
